# Age, Gender and Load-Related Influences on Left Ventricular Geometric Remodeling, Systolic Mid-Wall Function, and NT-ProBNP in Asymptomatic Asian Population

**DOI:** 10.1371/journal.pone.0156467

**Published:** 2016-06-09

**Authors:** Chi Chen, Kuo-Tzu Sung, Shou-Chuan Shih, Chuan-Chuan Liu, Jen-Yuan Kuo, Charles Jia-Yin Hou, Chung-Lieh Hung, Hung-I Yeh

**Affiliations:** 1 Department of Medicine, Mackay Medical College, New Taipei City, Taiwan; 2 Division of Cardiology, Department of Internal Medicine, Mackay Memorial Hospital, Taipei branch, Taipei City, Taiwan; 3 Division of Gastroenterology, Department of Internal Medicine, Mackay Memorial Hospital, Taipei branch, Taipei City, Taiwan; 4 The Institute of Health Policy and Management, College of Public Health, National Taiwan University, Taipei, Taiwan; University of Minnesota, UNITED STATES

## Abstract

**Background:**

Advanced age is associated with left ventricle (LV) remodeling and impaired cardiac function that may increase the risk of heart failure. Even so, studies regarding age-related cardiac remodeling in a large, asymptomatic Asian population remain limited.

**Materials and Methods:**

We studied 8,410 asymptomatic participants (49.7 ±11.7 y, 38.9% women) in a health evaluation cohort (2004–2012) at a tertiary center in Northern Taiwan. We analyzed age-related alterations for all echocardiography-derived cardiac structures/functions and the associations with circulating N-terminal prohormone of brain natriuretic peptide (NT-proBNP). We also explored sex-related differences in these measures.

**Results:**

In our cohort of 8,410 participants, advanced age was associated with greater LV wall thickness, larger LV total mass (+5.08 g/decade), and greater LV mass index (4.41 g/m^2^/decade), as well as increased serum NT-proBNP level (+18.89 pg/mL/decade). An accompanying reduction of stress-corrected midwall fractional shortening (–0.1%/decade) with aging was apparent in women after multi-variate adjustment (–0.09%/decade, *p* = 0.001). Furthermore, women demonstrated greater overall increase in LV wall thickness, LV mass index, and NT-proBNP compared to men (*p* for interaction: <0.001). All blood pressure components, including systolic, diastolic, and pulse pressures were independently associated with greater wall thickness and LV mass index after adjustment for confounders (all *p* <0.001). The associations between age and cardiac remodeling or mid-wall functions were further confirmed in a subset of study subjects with repeated follow up by GEE model.

**Conclusions:**

Significant associations of unfavorable LV remodeling and advanced age in our asymptomatic Asian population were observed, along with sex differences. These data may help explain the incidence of some diverse gender-related cardiovascular diseases, especially heart failure.

## Introduction

Age-related alterations in the cardiovascular system and heart functions have become increasingly crucial, in part because of the steady and rapid growth of the elderly population. Previous studies have demonstrated that altered cardiac structures and functions, such as concentric remodeling in terms of greater mass-to-volume ratio and diastolic dysfunction, may progress with aging [1–3]. These alterations can be explained in part by the accompanying elevation in load status with increasing age [4]. Phenotypic left ventricle (LV) hypertrophy is strongly associated with unfavorable cardiovascular events, especially heart failure and cardiovascular mortality [5, 6].

On the one hand, heart failure with preserved ejection fraction (HFpEF), which is closely associated with cardiac remodeling owing to advanced age and hypertension, has emerged as a new category of heart failure [7, 8]. On the other hand, several cross-sectional studies have explored sex differences in cardiac remodeling and HFpEF prevalence in Caucasian populations [4, 9–12]. However, data regarding such cardiac remodeling and functional changes with aging and possible gender differences remain relatively scarce with respect to Asian populations [13, 14]. A background knowledge and understanding of such information can be clinically meaningful and may contribute to a mechanistic understanding of how cardiac phenotypic structures or functions may adapt or remodel in response to senescence prior to HFpEF development in Asian patients [15].

Based on these considerations, we investigated age-related cardiac remodeling and functional changes in a large, asymptomatic Asian cohort. We further explored possible gender differences that could affect these functions.

## Materials and Methods

### Study Subjects

This study used a data set based on patients who underwent an annual cardiovascular examination at Mackay Memorial Hospital, a tertiary medical center at Northern Taipei, between July 2003 and December 2012. In total, 11,586 person-observations captured anthropometric measurements, body fat composition assessments, comprehensive echocardiography studies, 12-lead resting electrocardiography, and blood drawn for biochemical information and circulating biomarkers; all participants also completed structured health questionnaires. Among these patients, 9,058 had a baseline visit with nonrepeated echocardiography data. We precluded those for whom baseline variables were missing and those with a known implanted pacemaker, severe pulmonary hypertension (defined as peak systolic pulmonary artery pressure ≥60 mmHg), hypertrophic cardiomyopathy, atrial fibrillation, primary significant valvular heart diseases (aortic or mitral valves), or prevalent symptoms of heart failure (n = 648). All study participants showed preserved LV global ejection fraction (LVEF ≥50%). Some participants had repeated visits, and we used information from the first visit as representative data in this study. This cohort has been described and published in our previous study [16]. A subset of the studied subjects had multiple visits and echocardiography follow-ups. A total of 2,815 person-observations (1,910 subjects with second visit, and 873 had third or fourth visit) comprised the dataset for the longitudinal data analysis. We discarded 52 subjects whose visits occurred at irregular intervals within any single year, which comprised less than 5% of total person-observation numbers.

This study was approved by local ethical institutional committee (Mackay Memorial Hospital) for retrospective data analysis without informed consent of study participants (IRB No:14MMHIS245). Data security was guaranteed and all authors had no access to patient identifying information before and after data analysis. Study participants involved in this study were not under clinical service of current study physicians or researchers.

### Echocardiography

All study subjects who underwent transthoracic echocardiography were positioned in the left decubitus position after an adequate resting time. A Philips (Hewlett-Packard) Sonos 5500 ultrasound (Philips Ultrasound, Andover, MA, USA) was initially used for conventional echocardiography scans, and M-mode, two-dimensional (2D), and hemodynamic Doppler images were acquired according to a standardized protocol using a 2- to 4-MHz adult cardiac transducer (S4, phased array transducer). Since January 2009, echocardiography has been uniformly performed in our hospital using a GE system (Vivid i, Vingmed, Horten, Norway) equipped with 2- to 4-MHz transducer. For both systems, the standard echocardiography imaging protocol included M-mode measurement of left atrium diameter, LV internal diameters (LVID) at end-diastole (d) and end- systole (s) (LVIDd and LVIDs), wall thickness (IVS), and LV mass calculation (American Society of Echocardiography criteria), and LV volumes were determined by the modified biplane Simpson method by 2D. Left ventricular midwall fractional shortening (FSMMW) and stress-corrected midwall fractional shortening (FSCMW) were calculated by the following formulas [17]:

FSMMW={(LVIDd+IVSd/2+LVPWd/2)−(LVIDs+Hs/2)}/LVIDd+IVSd/2+LVPWd/2);

$$\text{FSCMW}=\left\{\left[\text{SBP}\times {\left(\text{LVIDs}/2\right)}^{2}]\times [1+{\left(\text{LVIDs}/2+\text{LV}-\text{LVPWs}\right)}^{2}/{\left(\text{LVIDs}/2+\text{LV}-\text{LVPWs}/2\right)}^{2}\right]\right\}/\{{\left(\text{LVIDs}/2+\text{LV}-\text{LVPWs}\right)}^{2}-{\left(\text{LVIDs}/2\right)}^{2}\},$$

where Hs and LVPW represent systolic thickness of the shell and LV posterior wall thickness. d and s represent cardiac phases at end-diastole and end-systole, respectively.

All M-mode images were acquired and recorded at a speed of 60–100 mm/s with the transducer placed at the third to fifth intercostal space. In a random set of 30 patients from the cohort, the intra- and inter-observer variability (coefficients of variance) for LV wall thickness measurements were 7.2% and 6.1%, respectively, and for diastolic diameter were 7.4% and 6.8%, respectively. Our study population comprised those with baseline visit demographics and those for whom non-repeated echocardiography data and M-mode LV geometric indices (such as LV wall thickness and diameters) were available for final statistical analysis (n = 8,410).

### Serum N-Terminal Prohormone of Brain Natriuretic Peptide Test and Renal Function Assessment

For study participants with baseline echocardiography data, additional data about N-terminal prohormone of brain natriuretic peptide (NT-ProBNP, pg/mL) were available for 6,061 study participants (72.1%) based on electrochemiluminescence immunoassay (Roche E170, Roche Diagnostics, Basel, Switzerland). Major baseline demographic information including age, sex distribution, body size, blood pressure, and medical histories did not differ significantly between study participants with or without NT-ProBNP data in our population. Renal function in terms of estimated glomerular filtration rate (eGFR) was assessed using the Modification of Diet in Renal Disease formula.

### Statistical Analysis

Continuous data were shown as means and standard deviations and were compared using the *t*-test; categorical data were expressed as the frequencies and proportions of data in all subjects and were compared using the chi-square test. A linear regression model was used to identify the associations between age and other LV geometric indices, which were initially assessed using a univariate model. Multi-variate models were then constructed to establish the independent relationships between age and LV geometric indices, including several clinical covariates such as sex, blood pressure components, body mass index (BMI), fasting glucose, cholesterol, high-density lipoprotein, renal function in terms of eGFR, and medical histories of hypertension, diabetes, cardiovascular diseases, or hyperlipidemia as confounders. Because of the potential collinearity among various blood pressure components (systolic blood pressure, diastolic blood pressure, and pulse pressure) in the associations with various LV geometric indices, these variables were included separately in multivariable models. Bivariate analysis was conducted to explore the associations between several LV geometric indices and NT-ProBNP levels, further adjusted by age and sex. To further clarify the role of aging on cardiac remodeling and to confirmed gender-related differences in a longitudinal fashion, uni-variate and multi-variate models were constructed using generalized estimating equations (GEE) in a subset of study cohort with repeated visits (additional n = 2815).

The *p* value was set using two-tailed probability, and a *p* value <0.05 was considered statistically significant. IBM SPSS version 22.0 (Chicago, IL, USA) and STATA 8.2 (StataCorp, College Station, TX, USA) were used to conduct the statistical analyses.

## Results

### Baseline Characteristics

Table 1 shows the baseline characteristics of the study participants, including all LV geometric data available in our study population (n = 8,410), sorted by genders. Generally, males were younger and had greater height, body weight, and waist and hip circumference (all *p* <0.001). Women showed lower blood pressure profiles (all *p* <0.001). Men tended to have higher fasting glucose levels, and women showed higher levels of high-density lipoprotein and eGFR (all *p* <0.001).

**Table 1:** Baseline characteristics and left ventricular conventional measures of all study participants (n = 8,410).

	Female (N = 3,272)	Male (N = 5,138)	P Value
Clinical characteristics			
Age, y	51.3 (12)	48.7 (11.4)	<0.001
Height, cm	157 (5.8)	170 (6.2)	<0.001
Weight, kg	56.8 (9.3)	71.9 (11.1)	<0.001
Body mass index	23.2 (3.7)	25 (3.4)	<0.001
SBP, mmHg	121 (19.4)	125 (16.4)	<0.001
DBP, mmHg	72.7 (10.8)	77.6 (10.8)	<0.001
MAP, mmHg	88.6 (12.7)	93.4 (11.6)	<0.001
PP, mmHg	47.9 (11.6)	47.3 (13.7)	0.034
Heart rate, beat/min	68.7 (15.1)	68.5 (12.5)	0.5692
Waist circumference, cm	75.1 (15.4)	85.6 (11.8)	<0.001
Hip circumference, cm	90.6 (16.1)	94.2 (10.8)	<0.001
Waist-to-hip ratio	0.83 (0.07)	0.91 (0.06)	<0.001
Cholesterol, mg/dL	200.4 (38)	198.8 (36)	0.058
Fasting glucose, mg/dL	98.4 (24.2)	102.7 (24.5)	<0.001
HDL, mg/dL	61.9 (15.7)	48.9 (12.1)	<0.001
eGFR, mg/dL	92.6 (20.3)	85.6 (15.8)	<0.001
Medical Histories, %			
Hypertension	638 (19.5%)	1005 (19.6%)	0.945
Hyperlipidemia	76 (2.3%)	129 (2.5%)	0.586
Diabetes mellitus	228 (7.0%)	345 (6.7%)	0.653
Cardiovascular disease	309 (9.4%)	395 (7.7%)	0.005
LeVentricular Conventional Indices			
IVS, mm	8.73 (1.13)	9.36 (0.96)	<0.001
LVPW, mm	8.72 (1.04)	9.34 (0.98)	<0.001
LVIDd, mm	44.6 (3.7)	47.9 (3.47)	<0.001
LVIDs, mm	27.8 (2.98)	30.2 (2.99)	<0.001
LV mass, g	127.6 (29.3)	156.3 (29.2)	<0.001
LV mass index	75.7 (15.8)	79.4 (14.1)	<0.001
FS, %	37.7 (4.04)	37 (3.97)	<0.001
FSCMW, %	22.7 (2.3)	22.4 (2.23)	<0.001
Ejection fraction, %	67.7 (5.11)	66.6 (5.2)	<0.001

Abbreviations: SBP, systolic blood pressure; DBP, diastolic blood pressure; MAP, mean arterial pressure; PP, pulse pressure; HDL, high-density lipoprotein; eGFR, Estimated Glomerular Filtration Rate; IVS, inter-ventricular septum; LVPW, left ventricularposterior wall; LVIDd, LV end-diastolic diameter; LVIDs, LV end-systolic diameter; FS, fractional shortening; FSCMW, stress-corrected mid-wall fractional shortening.

### Age-Related LV Structural Remodeling

**Fig 1:**
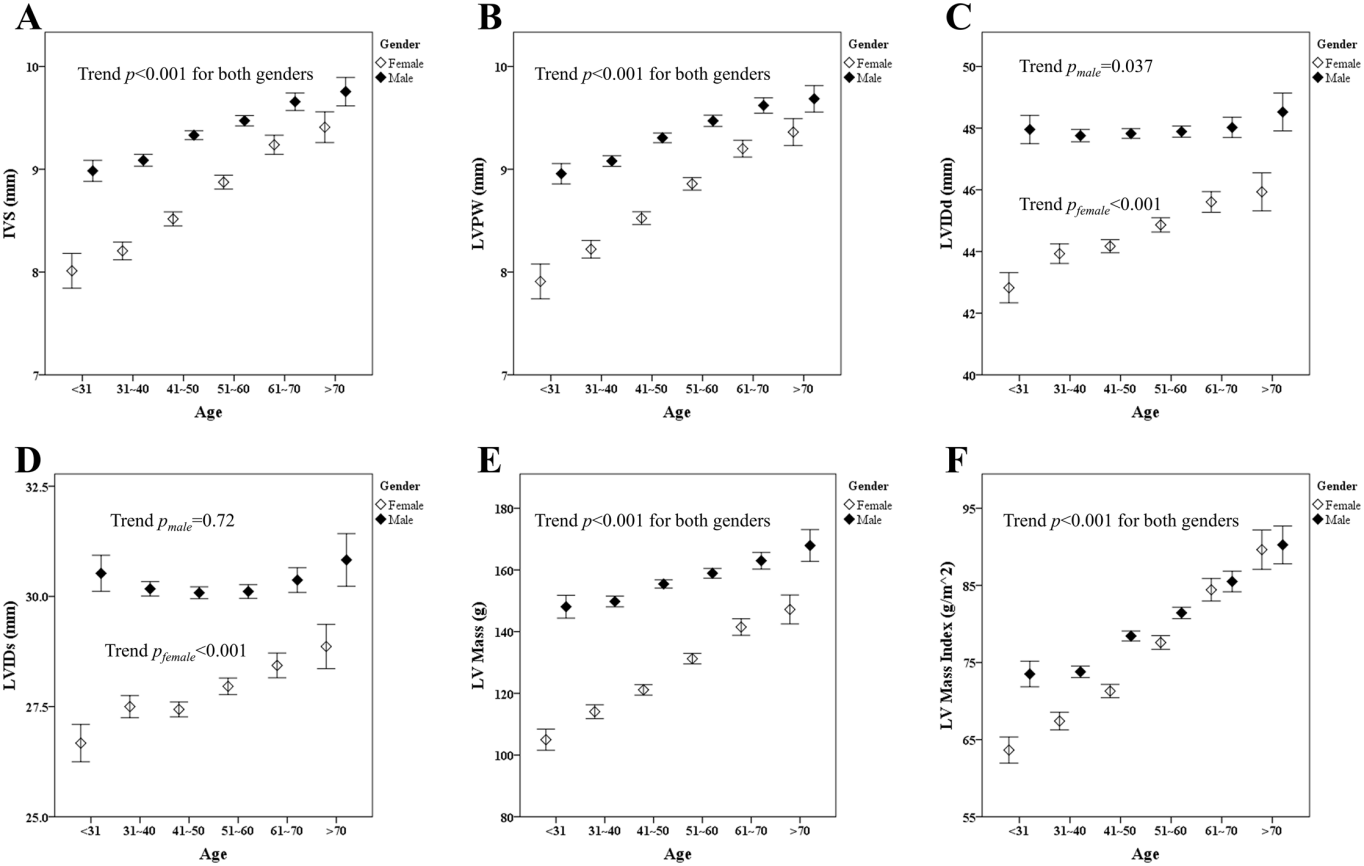
The means of interventricular septum (1A), left ventricle posterior wall (1B), left ventricle internal diameter end diastole (1C), left ventricle internal diameter end systole (1D), left ventricle mass (1E), and left ventricle mass index (1F) of different gender and age groups. The number of subjects in different age groups was 141, 464, 931, 1020, 515 and 201 for the following age groups: <30, 31~40, 41~50, 51~60, 61~70 and >71 (years) for female; and 253, 1011, 1724, 1430, 515, 205 for male as same age categories, respectively. All *p* values for trends are shown in the figure.

Fig 1 demonstrates that LV wall thicknesses, including the LV interventricular septum (IVS, Fig 1A) and LV posterior wall (LVPW, Fig 1B), increased with increasing age. Both measures of LV wall thickness were significantly larger with advanced age for both genders (trend *p* <0.001). A trend toward greater chamber diameter with more advanced age was observed only in women (Fig 1C and 1D, LVIDd and LVIDs, respectively), although, in general, chamber dimensions and wall thickness were larger in men than in women (Table 1). These differences resulted in a significantly greater LV mass (+28.7 g) and LV mass index (+3.7 g/m^2^) in men compared to those in women (both *p* <0.001). Similar trends were also found in participants without medical histories of hypertension, diabetes, cardiovascular disease, and hyperlipidemia (n = 6,093; age 47.1 ±10.9 y; 2,360 females [38.7%]; S1 Fig).

**Fig 2:**
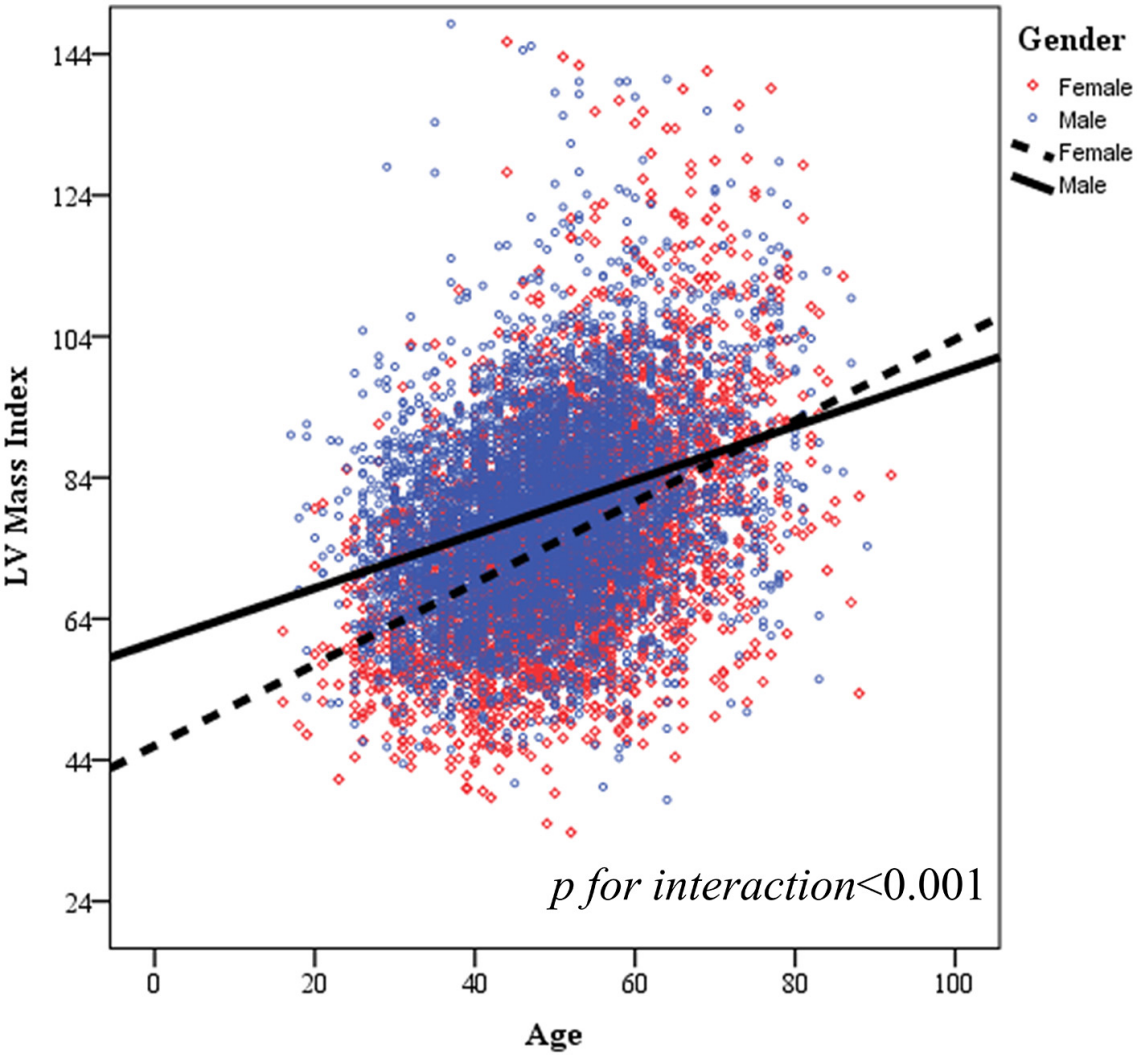
Scatter plot of age versus left ventricle mass index of both genders for all participants. In Fig 2, *p* for interaction: <0.001.

Table 2 shows the age-related distribution of LV mass and LV mass index for all participants (n = 8,410) stratified by gender (Tables 2 and 3 and Fig 1E and 1F). Both men and women demonstrated similar trends toward higher LV mass and LV mass index with more advanced age (+5.08 g/decade and +4.41 g/m^2^/decade for LV mass and LV mass index, respectively; both *p* <0.001), and men and women ≥70 y had LV mass indices that were nearly 13% and 40% greater, respectively, compared to those ≤30 y. The associations between greater LV mass index and increasing age remained unchanged in multi-variate models (Table 4). A significant interaction between genders in LV mass index with increasing age was observed (*p* <0.001, Fig 2), indicating that there is a steeper rise in LV mass index with advanced age in women compared with that in men (*p* for sex interaction <0.001). These associations were also found in healthy participants (S1 Table). Furthermore, higher blood pressure components were also related to greater LV mass in uni- (Coef.: 0.58, 0.91 and 0.48 per 1mmHg increase for systolic, diastolic and pulse pressure, respectively, all p<0.001) and multi-variate models for both genders (Table 4 and S2 Table, all p<0.05).

**Table 2:** The age-stratified value of LV mass in all study participants (n = 8,410).

LV Mass Data in All Study Participants (n = 8,410)				
Age Groups	All (n = 8,410)	Men	Women	p value¥
< = 30 (years)	Mean (SD)	148.1 (29.7)	105 (20.2)	<0.001
	n	253	141	
31~40 (years)	Mean (SD)	149.8 (26.8)	114 (23.8)	<0.001
	n	1011	464	
41~50 (years)	Mean (SD)	155.5 (27.4)	121.1 (25.7)	<0.001
	n	1724	931	
51~60 (years)	Mean (SD)	158.9 (29.6)	131.2 (27.3)	<0.001
	n	1430	1020	
61~70 (years)	Mean (SD)	163 (30.7)	141.5 (30.7)	<0.001
	n	515	515	
> = 71 (years)	Mean (SD)	167.9 (36.7)	198 (33.5)	<0.001
	n	205	201	
p for trend		<0.001	<0.001	
(across age groups)				
All subjects	Mean (SD)	156.3 (29.2)	127.5 (29.3)	<0.001
	n	5,138	3,272	

**Table 3:** The age-stratified value of LV mass index in all study participants (n = 8,410).

LV Mass Index Data in All Study Participants (n = 8,410)				
Age Groups	All (n = 8,410)	Men	Women	p value¥
< = 30 (years)	Mean (SD)	73.5 (13.3)	63.6 (10)	<0.001
	n	253	141	
31~40 (years)	Mean (SD)	73.8 (11.6)	67.4 (12)	<0.001
	n	1011	464	
41~50 (years)	Mean (SD)	78.4 (13)	71.3 (13.2)	<0.001
	n	1724	931	
51~60 (years)	Mean (SD)	81.4 (13.8)	77.6 (14.3)	<0.001
	n	1430	1020	
61~70 (years)	Mean (SD)	85.5 (15.2)	84.4 (16.7)	0.283
	n	515	515	
> = 71 (years)	Mean (SD)	90.3 (17.5)	89.6 (18.2)	0.727
	n	205	201	
p for trend		<0.001	<0.001	
(across age groups)				
All subjects	Mean (SD)	79.4 (14.1)	75.7 (15.8)	<0.001
	n	5,138	3,272	

**Table 4:** Association between age and LV indices in uni- and multi-variate models for all study participants (n = 8,410).

**Table 4A**									
**Age (per decade)**	**Female (n = 3,272)**			**Male (n = 5,138)**			**All study participants (n = 8,410)**		
Uni-variate model	Coef.		p	Coef.		p	Coef.		p
IVS	0.327		<0.001	0.185		<0.001	0.212		<0.001
LVPW	0.319		<0.001	0.18		<0.001	0.207		<0.001
LVIDd	0.613		<0.001	0.117		0.006	0.17		<0.001
LVIDs	0.401		<0.001	0.057		0.123	0.089		0.003
LV mass	9.14		<0.001	4.58		<0.001	5.08		<0.001
LV mass index	5.76		<0.001	3.83		<0.001	4.41		<0.001
FS	-0.036		0.539	0.045		0.356	0.043		0.246
FSMMW	-0.21		<0.001	-0.131		<0.001	-0.151		<0.001
FSCMW	-0.178		<0.001	-0.093		0.001	-0.111		<0.001
**Table 4B**									
**Age (per decade)**	**Female (n = 3,272)**			**Male (n = 5,138)**			**All study participants (n = 8,410)**		
Multi-variate Model 1	Coef.	p		Coef.	p		Coef.	p	
IVS	0.237	<0.001*		0.148	<0.001*		0.141	<0.001*	
LVPW	0.233	<0.001*		0.148	<0.001*		0.14	<0.001*	
LVIDd	0.376	<0.001*		0.033	0.443*		-0.046	0.225*	
LVIDs	0.255	<0.001*		0.038	0.793*		-0.049	0.112*	
LV mass	6.25	<0.001*		3.35	<0.001*		2.55	<0.001*	
LV mass index	4.66	<0.001*		3.45	<0.001*		3.66	<0.001*	
FS	-0.038	0.565		0.029	0.561		0.047	0.228	
FSMMW	-0.171	<0.001*		-0.124	<0.001		-0.127	<0.001*	
**Age (per decade)**	**Female (n = 3,272)**			**Male (n = 5,138)**			**All study participants (n = 8,410)**		
Multi-variate Model 2	Coef.	p		Coef.	p		Coef.	p	
IVS	0.203	<0.001*		0.141	<0.001*		0.125	<0.001*	
LVPW	0.204	<0.001*		0.14	<0.001*		0.124	<0.001*	
LVIDd	0.299	<0.001*		0.103	0.037*		-0.04	0.321*	
LVIDs	0.194	<0.001		0.034	0.437		-0.068	0.048*	
LV mass	5.3	<0.001*		3.5	<0.001*		2.27	<0.001*	
LV mass index†	4.76	<0.001*		3.32	<0.001*		3.52	<0.001*	
FS	-0.02	0.799		0.069	0.241*		0.098	0.044	
FSMMW	-0.147	<0.001		-0.088	0.003		-0.093	<0.001	
FSCMW‡	-0.12	0.005		-0.054	0.112		-0.056	0.03	

Coef.: beta co-efficiency; BMI: body mass index; FSMMW, mid-wall fractional shortening, other abbreviations as Table 1. Other abbreviations as Table 1.

Model 1: further adjusted for SBP;

Model 2: further adjusted for BMI, SBP, fasting glucose, total cholesterol, HDL, eGFR, medical histories of hypertension, diabetes, CVD, and hyperlipidemia;

^†^BMI not added in model,

^‡^SBP not added in the model.

*p value <0.05 for blood pressure component SBP;

Abbreviations as Table 1.

To be more precise about which structural alterations can be attributed to the observed LV mass changes, we also analyzed age-related alterations in cardiac geometric indices in our cohort. Overall, we found a trend toward greater cardiac geometric indices with advanced age for both genders except for LVIDs (Fig 1), and these independent associations remained unchanged for IVS and LVPW in multi-variate models (Table 4; all *p* <0.001). Further, a positive correlation between age and LVIDs was observed only in females (Fig 1 and Table 4). Interactions between gender and aging can be found in IVS, LVPW, LVIDd, and LVIDs (S2 Fig; all interactions *p* for sex; p <0.001). In addition, uni-variate models showed positive associations between all blood pressure components (systolic, diastolic and pulse pressure) and greater cardiac geometric indices (all p<0.001), and were further independently associated with increased IVS, LVPW, and LVIDd in multi-variate models for both genders (Table 4 and S2 Table).

#### Age-Related Alterations of LV Midwall Systolic Function Indicators

**Fig 3:**
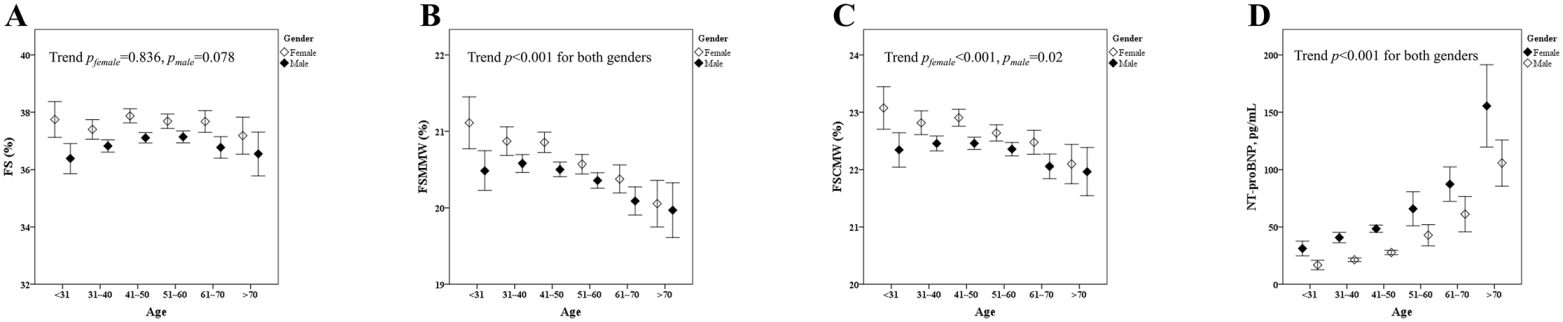
The mean values of fractional shortening (3A), midwall fractional shortening (3B), stress-corrected midwall fractional shortening (3C), and serum N-terminal prohormone of brain natriuretic peptide (3D) in different gender and age groups. Again, the number of subjects in different age groups was 141, 464, 931, 1020, 515 and 201 for the following age groups: <30, 31~40, 41~50, 51~60, 61~70 and >71 (years) for female; and 253, 1011, 1724, 1430, 515, 205 for male as same age categories, respectively. All *p* values for trends are shown in the figure.

To investigate the systolic function of left ventricle (LV) among different genders and age groups, we analyzed the alteration of fractional shortening (FS; Fig 3A). To overcome the potential limitations of masked myofiber dysfunction caused by geometric changes or load-related cardiac chamber adaptations [18–20], we further examined the associations between age, LV FSMMW and FSCMW, which represent electrocardiographic strain on LV midwall performances, in uni- and multi-variate models. (Fig 3B and 3C and S2 Table). In univariate models, both FSMMW and FSCMW decreased significantly with increasing age (adjusted estimate –0.2%/decade and –0.1%/decade; both *p* ≤0.001), although these independent associations with age remained constant in FSCMW only after accounting for other clinical covariates in women (*p* for sex interaction <0.001). Univariate analysis showed inverted associations between all BP components (systolic, diastolic and pulse pressure) and various LV midwall functions (all p<0.05) though we found that FS was not independently related to any blood pressure component in multi-variate models that included both sexes (Table 4 and S2 Table). Similar trends can also be found among healthy participants (S3 Fig). Some of the participants have repeated visits. We discarded repeated data in the same subject with more than 4 visits, which was quite few and contributed to less than 5% of total person-visit numbers. Additionally, 2815 person-visit data (totally 1910 subjects with second visits, and 873 had 3rd and 4th visit) were included in our longitudinal data analysis. In S5 Table, we further demonstrated similar trends in the associations between age and these cardiac remodeling or functional measures.

### Associations between NT-proBNP and Various LV Geometric Indices or Systolic Functional Indicators

Besides FSMMW and FSCMW, the serum level of NT-proBNP, a marker for subclinical LV dysfunction [21, 22], is significantly higher with advanced age (+18.89 pg/mL/decade) (Fig 3D). Again, this association is more pronounced in women (*p* for sex interaction 0.001). We also found associations among NT-proBNP level, LV mass, LV mass index, and other LV geometric indices between genders in bivariate and age- and sex-adjusted models (S3 Table). Unfavorable LV geometric alterations or remodeling, including LV diameter or total mass rather than chamber-based systolic indices such as FS or FSCMW, were more tightly linked to elevated NT-proBNP in our cohort (all adjusted *p* <0.001).

## Discussion

In this retrospective analysis, we found that several echocardiographic indices, including LV wall thickness, total LV mass, and LV mass index all increased with age in a large asymptomatic Asian cohort. These structural alterations remain unchanged even after we accounted for several clinical factors. We also observed an age-related differential cardiac remodeling pattern between the two sexes. Although women demonstrate a larger slope of LV mass increase with aging compared to men, there is also a trend toward age-related attenuation of LV systolic midwall function in women that can be partially explained by a more pronounced increase in LV chamber size among elderly women.

Both LV mass and LV mass index increased steadily with age in our cohort, and these findings are consistent with those of previous studies in Caucasian and Asian populations [9, 10, 13, 14]. As a matter of fact, our data suggest that both men and women >65 y have nearly 19.8 g and 42.2 g higher LV mass, respectively, than do those ≤35 y. Increased LV mass and LV mass index have been proposed as powerful predictors for the incidence and prognosis of several cardiovascular diseases, including heart failure, coronary artery disease, and angina pectoris, in both high-risk and general populations even with adjustment for age [7, 8, 23–27]. In addition, some have proposed that such alterations in LV geometry, mostly indicated by excessive LV total mass, are associated with worse LV chamber function in terms of reduced stress-corrected LV midwall shortening [28]. In this study, we found that several aspects of structural remodeling, especially LV wall thickness, chamber size, and LV mass index, were tightly linked to blood NT-proBNP levels. Such findings not only support previous studies but also facilitate the establishment of reliable reference values for these indices among Asian individuals in different age groups. This, in turn, may help clinicians in their routine evaluations of echocardiographs.

Although LV masses are significantly higher in elderly groups of both genders, younger women have a notably larger increase of LV mass and LV mass index than men do. Effects of sex-related hormonal changes with increasing age may in part account for this phenomenon, in particular for elderly women who probably have experienced menopause and have low estrogen levels. Estrogen has significant effects on the cardiovascular system, and functional estrogen receptors can be found in the ventricular myocardium, where it can affect the cellular physiology of cardiac tissues both genomically and nongenomically [29]. Within the female population, menopause is significantly associated with higher LV mass, LV mass index, and IVS thickness after adjustment for age, blood pressure, BMI, and other clinical covariates (S4 Table), indicating that sex hormones may play an important role in unfavorable ventricular remodeling.

Because the equation used to calculate LV mass is composed of LVIDd, LVPW, and IVS [30], we analyzed the changes of these indices among different age groups. In general, LV wall thicknesses, including LVPW and IVS, increase with advanced age, but the trend toward greater cardiac diameters (such as LVIDd and LVIDs) can be found only in women. These data may imply that age-related LV remodeling in females is primarily driven by both increased LV internal dimensions and wall thickness, but in males such age-associated increases in LV wall thickness could be the primary cause for LV mass increase. This can probably be ascribed to a more prominent increase of LV total mass in women as they age because both cardiac wall thickness and diameter may contribute to LV total mass increase with aging. Whether these effects of wall thickness increase and chamber dilation together are relevant to gender-specific hormones may require further investigation. So far, age-related discrepancies in LV chamber or diameter changes between genders have been described in previous studies [31–34]. Some of these studies are in concordance with our data and suggest that women tend to have larger LV chamber dimensions with aging, although other results are not consistent with our findings. We speculate that these diverse findings could be the results of ethnic diversity. A recent study that compared multiple ethnic groups, including Europeans, Asians, and Africans, suggested that not only the trends toward altered LVIDd but also the upper reference values are different among these groups [31]. Such racial disparities may cause different risks for cardiovascular diseases in these populations [14, 15]. Previous large epidemiological or observational studies have indicated that elderly women have higher risks of HFpEF, but men have a higher risk of heart failure associated with reduced ejection fraction [7, 8]. Moreover, LV hypertrophy is an independent predictor of all-cause death in patients with HFpEF [7].

Emerging data had demonstrated that subjects with HFpEF were typically featured by certain phenotypic remodeling including increased wall thickness, greater total LV mass, and worsened LV mid-wall function [35, 36]. Nevertheless, the presence of phenotype diversity (eg. Normal geometry or eccentric form of LVH) from population studies since 2007 had complicated the clinical diagnosis of HFpEF simply by describing LV morphology [37, 38]. Advanced age and female gender are two major influencing factors for the development of HFpEF together with several co-morbidities, including hypertension or obesity [36]. So far, data about the clinical risk factors of HFpEF and its predominant cardiac phenotypes from large Asian cohorts are relative limited. In one relatively large Asian ethnic research comparing LV pressure-volume associations in HFpEF, greater chamber size (eccentricity) in HFpEF as compared to asymptomatic HTN patients had been reported [39]. Age-related cardiac remodeling with a tendency for greater LV diameter in women had also been observed in another Asian cohort. [40] Interestingly, these data were similar to the trend of age- and load-related cardiac structural/functional alterations in our current work. In addition, another recent study by Li et al also demonstrated that elderly women may have greater chance to develop eccentric form LVH compared to men in a relatively large (n = 9,286) hypertensive Asians [41]. These data was consistent to our findings in gender-specific differences of cardiac remodeling with aging or under excessive load, and fitted our hypothetical model of HFpEF phenotypes in subjects under relevant risks.

Taken together, we therefore hypothesized that our findings may represent a certain cardiac remodeling with increasing age and excessive load at a pre-clinical phase, which might be the putative precursor for subsequent HFpEF development. In addition, there was a trend toward higher degree of cardiac remodeling in elderly women than men, which recapitulated most observed epidemiological findings of HFpEF. Notably, whether our findings are specific to all Asians or maybe applicable to a certain sub-group in Asian population remains yet unknown, and may need future large-scale survey to explore relevant issues.

### Study Limitations

Several limitations of this study should be noted. First, the cross-sectional study design limits observations of causal relationships among older age and LV mass and other relevant indices and risk factors. Second, this is a single-center study. As a result, it may not represent community data, and studies in different populations are needed.

## Conclusions

To the best of our knowledge, this is the largest cross-sectional study that compares and explores the correlations among age, LV geometric alterations, and systolic midwall functions in an Asian population and that concurrently considers sex-related differences in the data. According to our findings, LV may remodel in certain ways in association with advanced age, primarily resulting in increased cardiac wall thickness and greater LV total mass. In addition, unlike corresponding processes in Caucasian populations, ventricular remodeling among Asian females may differ from that of males by involving greater cardiac chamber size with advanced age and thereby resulting in a more pronounced rise of LV mass. Further research is needed to explore the causes of racial differences in age- and sex-related LV remodeling and to show how these remodeling processes may influence patients’ clinical risk of cardiovascular diseases, especially heart failure, in the elderly.

## Supporting Information

S1 Fig The mean of IVS (1A), LVPW (1B), LVIDd (1C), LVIDs (1D), LV mass (1E), LV mass index (1F) of participants without medical histories of hypertension, diabetes, cardiovascular disease, and hyperlipidemia in different gender and age groups (n = 6,093). All p for trend are marked on the figure.
(TIF)

S2 Fig The mean of FS (2A), FSMMW (2B), FSCMW (2C), serum NT-proBNP (2D) of of participants without medical histories of hypertension, diabetes, cardiovascular disease, and hyperlipidemia in different gender and age groups (n = 6,093). All p for trend are marked on the figure.
(TIF)

S3 Fig The scatter plot of age versus IVS (Sup 3A), LVPW (Sup 3B), LVIDd (Sup 3C) and LVIDS (Sup 3D) of both gender for all participants. All p for interaction <0.001.
(TIF)

S1 Table The associations between age, load status and LV mass index in uni- and multi-variate models stratified by sex for healthy participants (n = 6,093). (DOC)

S2 Table Associations between age and several LV geometric indices in multi-variate models adjusted with DBP or PP for all study participants (n = 8,410). (DOC)

S3 Table The associations between NT-proBNP and LV mass, LV mass index, LV wall thickness, fractional shortening (FS) and stress-corrected mid-wall fractional shortening (FSCMW) (n = 6,123). (DOC)

S4 Table The associations between Menopause Status and LV mass measure in age-, BMI- and SBP-adjusted multi-variate models for women (n = 3,272). (DOC)

S5 Table Association between age and LV indices in uni- and multi-variate models for all study participants in GEE models (n = 11,225). (DOC)
