# Supplementary material for: Age, Gender and Load-Related Influences on Left Ventricular Geometric Remodeling, Systolic Mid-Wall Function, and NT-ProBNP in Asymptomatic Asian Population
Source: PLoS One. 2016 Jun 9;11(6):e0156467. doi: 10.1371/journal.pone.0156467 (PMC4900638; doi:10.1371/journal.pone.0156467)
Supplement: S1 Table — (DOC) [file pone.0156467.s004.doc]

**S1 Table. The associations between age, load status and LV mass index in uni- and multi-variate models stratified by sex for healthy participants (n=6,093)**

|  |  | **LV mass index, gm/m^2^** | | | | | |
| --- | --- | --- | --- | --- | --- | --- | --- |
| Uni-variate model | | Female (n=2,361) | | Male (n=3,732) | | All healthy participants (n=6,093) | |
|  | | Coef. | p | Coef. | p | Coef. | p |
| Age (per decade) | | 4.94 | <0.001 | 3.36 | <0.001 | 3.76 | <0.001 |
|  | |  | | | | | |
|  | | **LV mass index, gm/m^2^** | | | | | |
| Multi-variate models | | Female (n=2,361) | | Male (n=3,732) | | All study participants (n=6,093) | |
|  | | Coef. | p | Coef. | p | Coef. | p |
| Model 1 | |  |  |  |  |  |  |
| Age (per decade) | | 4.34 | <0.001 | 3.15 | <0.001 | 3.3 | <0.001 |
| SBP (per 10mmHg) | | 1.04 | <0.001 | 0.98 | <0.001 | 1.4 | <0.001 |
| Model 2 | |  |  |  |  |  |  |
| Age (per decade) | | 4.14 | <0.001 | 3.13 | <0.001 | 3.33 | <0.001 |
| SBP (per 10mmHg) | | 0.72 | <0.001 | 1.01 | <0.001 | 1.15 | <0.001 |
| BMI (per 5kg/m^2^) | | 2.67 | <0.001 | -0.283 | 0.390 | 1.75 | <0.001 |
| Model 3* | |  |  |  |  |  |  |
| Age (per decade) | | 4.68 | <0.001 | 3.59 | <0.001 | 3.6 | <0.001 |
| SBP (per 10mmHg) | | 0.57 | 0.005 | 0.99 | <0.001 | 1.08 | <0.001 |
| BMI (per 5kg/m^2^) | | 2.54 | <0.001 | 0.11 | 0.759 | 1.36 | <0.001 |

|  |  | **LV mass index, gm/m^2^** | | | | | |
| --- | --- | --- | --- | --- | --- | --- | --- |
| Uni-variate model | | Female (n=2,361) | | Male (n=3,732) | | All healthy participants (n=6,093) | |
|  | | Coef. | p | Coef. | p | Coef. | p |
| Age (per decade) | | 4.94 | <0.001 | 3.36 | <0.001 | 3.76 | <0.001 |
|  | |  | | | | | |
|  | | **LV mass index, gm/m^2^** | | | | | |
| Multi-variate models | | Female (n=2,361) | | Male (n=3,732) | | All healthy participants (n=6,093) | |
|  | | Coef. | p | Coef. | p | Coef. | p |
| Model 1 | |  |  |  |  |  |  |
| Age (per decade) | | 4.55 | <0.001 | 3.26 | <0.001 | 3.52 | <0.001 |
| DBP (per 10mmHg) | | 1.63 | <0.001 | 1.08 | <0.001 | 1.97 | <0.001 |
| Model 2 | |  |  |  |  |  |  |
| Age (per decade) | | 4.28 | <0.001 | 3.26 | <0.001 | 3.52 | <0.001 |
| DBP (per 10mmHg) | | 1.16 | <0.001 | 1.12 | <0.001 | 1.57 | <0.001 |
| BMI (per 5kg/m^2^) | | 2.69 | <0.001 | -0.18 | 0.587 | 1.79 | <0.001 |
| Model 3* | |  |  |  |  |  |  |
| Age (per decade) | | 4.78 | <0.001 | 3.69 | <0.001 | 3.77 | <0.001 |
| DBP (per 10mmHg) | | 1.07 | 0.001 | 1.28 | <0.001 | 1.58 | <0.001 |
| BMI (per 5kg/m^2^) | | 2.49 | <0.001 | 0.15 | 0.672 | 1.36 | <0.001 |

|  |  | **LV mass index, gm/m^2^** | | | | | |
| --- | --- | --- | --- | --- | --- | --- | --- |
| Uni-variate model | | Female (n=2,361) | | Male (n=3,732) | | All healthy participants (n=6,093) | |
|  | | Coef. | p | Coef. | p | Coef. | p |
| Age (per decade) | | 4.94 | <0.001 | 3.36 | <0.001 | 3.76 | <0.001 |
|  | |  | | | | | |
|  | | **LV mass index, gm/m^2^** | | | | | |
| Multi-variate models | | Female (n=2,361) | | Male (n=3,732) | | All healthy participants (n=6,093) | |
|  | | Coef. | p | Coef. | p | Coef. | p |
| Model 1 | |  |  |  |  |  |  |
| Age (per decade) | | 4.71 | <0.001 | 3.23 | <0.001 | 3.54 | <0.001 |
| PP (per 10mmHg) | | 0.69 | 0.007 | 0.98 | <0.001 | 1.09 | <0.001 |
| Model 2 | |  |  |  |  |  |  |
| Age (per decade) | | 4.36 | <0.001 | 3.23 | <0.001 | 3.52 | <0.001 |
| PP (per 10mmHg) | | 0.43 | 0.099 | 0.98 | <0.001 | 0.88 | <0.001 |
| BMI (per 5kg/m^2^) | | 3.02 | <0.001 | 0.13 | 0.690 | 2.37 | <0.001 |
| Model 3* | |  |  |  |  |  |  |
| Age (per decade) | | 4.88 | <0.001 | 3.67 | <0.001 | 3.75 | <0.001 |
| PP (per 10mmHg) | | 0.22 | 0.411 | 0.77 | <0.001 | 0.72 | <0.001 |
| BMI (per 5kg/m^2^) | | 2.81 | <0.001 | 0.51 | 0.154 | 1.85 | <0.001 |

Abbreviations as Table 1 and Table 3.

Healthy participants: participants without medical histories of hypertension, diabetes, CVD, and hyperlipidemia.

BMI: body mass index, SBP: systolic blood pressure.

*Model 3: CV indicates other clinical variables including fasting glucose, cholesterol, HDL, eGFR,
