# Supplementary material for: Age, Gender and Load-Related Influences on Left Ventricular Geometric Remodeling, Systolic Mid-Wall Function, and NT-ProBNP in Asymptomatic Asian Population
Source: PLoS One. 2016 Jun 9;11(6):e0156467. doi: 10.1371/journal.pone.0156467 (PMC4900638; doi:10.1371/journal.pone.0156467)
Supplement: S2 Table — (DOC) [file pone.0156467.s005.doc]

**S2 Table. Associations between age and several LV geometric indices in multi-variate models adjusted with DBP or PP for all study participants (n=8,410)**

Supplementary Table 2A

| **Age (per decade)** | **Female (n=3,272)** | | | **Male (n=5,138)** | | **All study participants (n=8,410)** | |
| --- | --- | --- | --- | --- | --- | --- | --- |
| Multi-variate Model 1 | Coef. | | p | Coef. | p | Coef. | p |
| IVS | 0.278 | | <0.001* | 0.171 | <0.001* | 0.179 | <0.001* |
| LVPW | 0.275 | | <0.001* | 0.168 | <0.001* | 0.177 | <0.001* |
| LVIDd | 0.487 | | <0.001* | 0.093 | 0.028* | 0.075 | 0.036* |
| LVIDs | 0.31 | | <0.001* | 0.045 | 0.219* | 0.026 | 0.385* |
| LV mass | 6.25 | | <0.001* | 3.35 | <0.001* | 2.55 | <0.001* |
| LV mass index | 5.23 | | <0.001* | 3.74 | <0.001* | 4.15 | <0.001* |
| FS | -0.009 | | 0.886 | 0.038 | 0.433 | 0.052 | 0.168 |
| FSMMW | -0.18 | | <0.001* | -0.127 | <0.001 | -0.138 | <0.001* |
|  |  | | |  | |  | |
| **Age (per decade)** | **Female (n=3,272)** | | | **Male (n=5,138)** | | **All study participants (n=8,410)** | |
| Multi-variate Model 2 | Coef. | p | | Coef. | p | Coef. | p |
| IVS | 0.214 | <0.001* | | 0.152 | <0.001* | 0.142 | <0.001* |
| LVPW | 0.216 | <0.001* | | 0.15 | <0.001* | 0.14 | <0.001* |
| LVIDd | 0.325 | <0.001 | | 0.125 | 0.011 | 0.003 | 0.932* |
| LVIDs | 0.029 | 0.506 | | 0.207 | <0.001 | -0.053 | 0.125* |
| LV mass | 5.3 | <0.001* | | 3.5 | <0.001* | 2.27 | <0.001* |
| LV mass index† | 5.04 | <0.001* | | 3.51 | <0.001* | 3.82 | <0.001* |
| FS | 0.014 | 0.847 | | 0.085 | 0.147* | 0.106 | 0.018 |
| FSMMW | -0.142 | <0.001 | | -0.085 | 0.004 | -0.093 | <0.001* |
| FSCMW‡ | -0.12 | 0.005 | | -0.054 | 0.112 | -0.056 | 0.03 |
| Model 1: further adjusted for DBP; | | | | | | | |
| Model 2: further adjusted for BMI, DBP, fasting glucose, total cholesterol, HDL, eGFR, medical histories of hypertension, diabetes, CVD, and hyperlipidemia; | | | | | | | |
| †BMI not added in model, ‡DBP not added in the model; | | | | | | | |
| * *p* value <0.05 for blood pressure component DBP;  Abbreviations as Table 1 and Table 3. | | | | | | | |

**Supplementary Table 2B**

| **Age (per decade)** | **Female (n=3,272)** | | **Male (n=5,138)** | | **All study participants (n=8,410)** | |
| --- | --- | --- | --- | --- | --- | --- |
| Multi-variate Model 1 | Coef. | p | Coef. | p | Coef. | p |
| IVS | 0.278 | <0.001* | 0.167 | <0.001* | 0.18 | <0.001* |
| LVPW | 0.27 | <0.001* | 0.163 | <0.001* | 0.174 | <0.001* |
| LVIDd | 0.483 | <0.001* | 0.052 | 0.232* | 0.063 | 0.1* |
| LVIDs | 0.338 | <0.001* | 0.016 | 0.671* | 0.026 | 0.414* |
| LV mass | 6.25 | <0.001* | 3.35 | <0.001* | 2.55 | <0.001* |
| LV mass index | 5.08 | <0.001* | 3.51 | <0.001* | 3.9 | <0.001* |
| FS | -0.075 | 0.251 | 0.039 | 0.438 | 0.03 | 0.449 |
| FSMMW | -0.202 | <0.001 | -0.129 | <0.001 | -0.144 | <0.001 |
|  |  | |  | |  | |
| **Age (per decade)** | **Female (n=3,272)** | | **Male (n=5,138)** | | **All study participants (n=8,410)** | |
| Multi-variate Model 2 | Coef. | p | Coef. | p | Coef. | p |
| IVS | 0.213 | <0.001 | 0.148 | <0.001* | 0.136 | <0.001* |
| LVPW | 0.211 | <0.001* | 0.144 | <0.001* | 0.133 | <0.001* |
| LVIDd | 0.304 | <0.001 | 0.098 | 0.046* | -0.023 | 0.578* |
| LVIDs | 0.029 | 0.506 | 0.207 | <0.001 | -0.053 | 0.125 |
| LV mass | 5.3 | <0.001* | 3.5 | <0.001* | 2.27 | <0.001* |
| LV mass index† | 4.93 | <0.001* | 3.33 | <0.001* | 3.59 | <0.001* |
| FS | -0.04 | 0.6* | 0.071 | 0.23 | 0.081 | 0.077 |
| FSMMW | -0.164 | <0.001 | -0.091 | 0.002 | -0.101 | <0.001 |
| FSCMW‡ | -0.12 | 0.005 | -0.054 | 0.112 | -0.056 | 0.03 |
| Model 1: further adjusted for PP | | | | | | |
| Model 2: further adjusted for BMI, PP, fasting glucose, total cholesterol, HDL, eGFR, medical histories of hypertension, diabetes, CVD, and hyperlipidemia | | | | | | |
| †BMI not added in model, ‡PP not added in the model.  * *p* value <0.05 for blood pressure component PP;  Abbreviations as Table 1 and Table 3. | | | | | | |
|  | | | | | | |
