# Supplementary material for: Age, Gender and Load-Related Influences on Left Ventricular Geometric Remodeling, Systolic Mid-Wall Function, and NT-ProBNP in Asymptomatic Asian Population
Source: PLoS One. 2016 Jun 9;11(6):e0156467. doi: 10.1371/journal.pone.0156467 (PMC4900638; doi:10.1371/journal.pone.0156467)
Supplement: S3 Table — (DOC) [file pone.0156467.s006.doc]

**S3 Table. The associations between NT-proBNP and LV mass, LV mass index, LV wall thickness, fractional shortening (FS) and stress-corrected mid-wall fractional shortening (FSCMW) (n=6,123)**

| All participants (n=6,061) | | | | | |
| --- | --- | --- | --- | --- | --- |
| Uni-variate | Coef. | p | Age, Sex-adjusted | Coef. | p |
| IVS, mm | 0.062 | <0.001 | IVS (mm) | 0.05 | 0.001 |
| LVPW, mm | 0.047 | 0.001 | LVPW (mm) | 0.04 | 0.003 |
| LVIDd, mm | 0.071 | <0.001 | LVIDd (mm) | 0.11 | <0.001 |
| LVIDs, mm | 0.04 | 0.006 | LVIDs (mm) | 0.08 | <0.001 |
| LV Mass, gm | 0.087 | <0.001 | LV Mass (g) | 0.12 | <0.001 |
| LV Mass Index, gm/m^2^ | 0.107 | <0.001 | LV Mass Index | 0.15 | <0.001 |
| FS, % | 0.066 | 0.027 | FS (%) | 0.001 | 0.923 |
| FSCMW, % | 0.028 | 0.064 | FSCMW (%) | 0.02 | 0.107 |

Abbreviations as Table 1 and Table 3.
