# Supplementary material for: Age, Gender and Load-Related Influences on Left Ventricular Geometric Remodeling, Systolic Mid-Wall Function, and NT-ProBNP in Asymptomatic Asian Population
Source: PLoS One. 2016 Jun 9;11(6):e0156467. doi: 10.1371/journal.pone.0156467 (PMC4900638; doi:10.1371/journal.pone.0156467)
Supplement: S4 Table — (DOC) [file pone.0156467.s007.doc]

**S4 Table. The associations between Menopause Status and LV mass measure in age-, BMI- and SBP-adjusted multi-variate models for women (n=3,272)**

| Uni-variate (age per decade) | Menopause Status (n=3,272) | | Multi-variate (age + BMI + SBP) Menopause Status (n=3,272) | | Multi-variate (age + BMI + SBP + CV) Menopause Status (n=3,272) | |
| --- | --- | --- | --- | --- | --- | --- |
|  | Coef. | p | Coef. | p | Coef. | p |
| IVS (mm) | 0.49 | <0.001 | 0.15 | <0.001 | 0.108 | 0.047 |
| LVPW (mm) | 0.46 | <0.001 | 0.11 | 0.003 | 0.081 | 0.106 |
| LVIDd (mm) | 0.67 | <0.001 | 0.12 | 0.276 | 0.098 | 0.603 |
| LVIDs (mm) | 0.47 | <0.001 | 0.10 | 0.03 | 0.123 | 0.433 |
| LV Mass (g) | 13.48 | <0.001 | 3.80 | <0.001 | 3.143 | 0.016 |
| LV Mass Index† | 8.53 | <0.001 | 2.58 | <0.001 | 1.988 | 0.009 |

Abbreviations as Table 1 and Table 3.

CV: fasting glucose, total cholesterol, HDL, eGFR, medical histories of hypertension, diabetes, CVD, and hyperlipidemia.

† BMI not added in multi-variate model
