# Supplementary material for: Age, Gender and Load-Related Influences on Left Ventricular Geometric Remodeling, Systolic Mid-Wall Function, and NT-ProBNP in Asymptomatic Asian Population
Source: PLoS One. 2016 Jun 9;11(6):e0156467. doi: 10.1371/journal.pone.0156467 (PMC4900638; doi:10.1371/journal.pone.0156467)
Supplement: S5 Table — (DOC) [file pone.0156467.s008.doc]

| **S5 Table. Association between age and LV indices in uni- and multi-variate models for all study participants in GEE models (n=11,225)** | | | | | | |
| --- | --- | --- | --- | --- | --- | --- |
| **Table 5A** |  |  |  |  |  |  |
| **Age (per decade)** | **Female (n=4,239)** | | **Male (n=6,982)** | | **All study participants (n=11,225)** | |
| *GEE model (Uni-variate)* | *Coef.* | *p* | *Coef.* | *p* | *Coef.* | *p* |
| IVS, mm | 0.329 | <0.001 | 0.175 | <0.001 | 0.213 | <0.001 |
| LVPW, mm | 0.319 | <0.001 | 0.177 | <0.001 | 0.211 | <0.001 |
| LVIDd, mm | 0.582 | <0.001 | 0.097 | 0.014 | 0.17 | <0.001 |
| LVIDs, mm | 0.397 | <0.001 | 0.085 | 0.015 | 0.12 | <0.001 |
| LV mass, gm | 9.09 | <0.001 | 4.57 | <0.001 | 5.30 | <0.001 |
| LV mass index, gm/m^2^ | 5.71 | <0.001 | 3.85 | <0.001 | 4.43 | <0.001 |
| FS, % | -6.97 | 0.182 | -0.04 | 0.368 | -0.02 | 0.518 |
| FSMMW, % | -0.24 | <0.001 | -0.18 | <0.001 | -0.19 | <0.001 |
| FSCMW, % | -0.21 | <0.001 | -0.15 | <0.001 | -0.16 | <0.001 |
| **Table 5B** |  |  |  |  |  |  |
| **Age (per decade)** | **Female (n=4,239)** | | **Male (n=6,982)** | | **All study participants (n=11,225)** | |
| *GEE model (Multi-variate)* | *Coef.* | *p* | *Coef.* | *p* | *Coef.* | *p* |
| IVS, mm | 0.20 | <0.001 | 0.14 | <0.001 | 0.164 | <0.001 |
| LVPW, mm | 0.19 | <0.001 | 0.14 | <0.001 | 0.162 | <0.001 |
| LVIDd, mm | 0.19 | 0.022 | 0.16 | 0.076 | 0.199 | 0.005 |
| LVIDs, mm | 0.21 | 0.061 | 0.001 | 0.991 | 0.09 | 0.144 |
| LV mass, gm | 4.73 | <0.001 | 4.23 | <0.001 | 4.45 | <0.001 |
| LV mass index, gm/m^2^† | 4.03 | <0.001 | 3.34 | <0.001 | 3.63 | <0.001 |
| FS, % | -0.18 | 0.177 | 0.22 | 0.022 | 0.073 | 0.356 |
| FSMMW, % | -0.23 | 0.002 | -0.02 | 0.709 | -0.09 | 0.022 |
| FSCMW, %‡ | -0.22 | 0.004 | 0.02 | 0.772 | -0.08 | 0.81 |
| Abbreviations as Table 1 and Table 3. | | | | | | |
| GEE model further adjusted for BMI, SBP, fasting glucose, total cholesterol, HDL, eGFR, medical histories of hypertension, diabetes, CVD, and hyperlipidemia | | | | | | |
| †BMI not added in model, ‡BP components not added in the model | | | | | | |
| BMI: body mass index, SBP, systolic blood pressure. | | | | | | |
